# Influence of Belatacept- vs. CNI-Based Immunosuppression on Vascular Stiffness and Body Composition

**DOI:** 10.3390/jcm11051219

**Published:** 2022-02-24

**Authors:** Zbigniew Heleniak, Sarah Illersperger, Marcel G. Naik, Bilgin Osmanodja, Simon Ronicke, Georgios Eleftheriadis, Fabian Halleck, Klemens Budde

**Affiliations:** 1 Department of Nephrology, Transplantology and Internal Medicine, Medical University of Gdansk, 7 Dębinki Street, 80-952 Gdańsk, Poland; 2 Medizinische Klinik mit Schwerpunkt Nephrologie und Internistische Intensivmedizin, Charité–Universitätsmedizin, 10117 Berlin, Germany; sarah.illersperger@charite.de (S.I.); marcel.naik@charite.de (M.G.N.); bilgin.osmanodja@charite.de (B.O.); simon.ronicke@charite.de (S.R.); georgios.eleftheriadis@charite.de (G.E.); fabian.halleck@charite.de (F.H.); klemens.budde@charite.de (K.B.)

**Keywords:** kidney transplantation, belatacept, arterial stiffness

## Abstract

Background: Arterial stiffness and phase angle (PhA) have gained importance as a diagnostic and prognostic parameter in the management of cardiovascular disease. There are few studies regarding the differences in arterial stiffness and body composition between renal transplant recipients (RTRs) receiving belatacept (BELA) vs. calcineurin inhibitors (CNI). Therefore, we investigated the differences in arterial stiffness and body composition between RTRs treated with different immunosuppressants, including BELA. Methods: In total, 325 RTRs were enrolled in the study (mean age 52.2 years, M −62.7%). Arterial stiffness was determined with an automated oscillometric device. All body composition parameters were assessed, based on bioelectrical impedance analysis (BIA), and laboratory parameters were obtained from the medical files of the patients. Results: We did not detect any significant difference in terms of arterial stiffness and PhA in RTRs undergoing different immunosuppressive regimens, based on CsA, Tac, or BELA. Age was an essential risk factor for greater arterial stiffness. The PhA was associated with age, BMI, time of dialysis before transplantation, and kidney graft function. Conclusion: No significant differences in arterial stiffness and PhA were observed in RTRs under different immunosuppressive regimens. While our data provide additional evidence for arterial stiffness and PhA in RTRs, more research is needed to fully explore these cardiovascular risk factors and the impact of different immunosuppressive regimens.

## 1. Introduction

### 1.1. Cardiovascular Disease after Kidney Transplantation

Kidney transplantation (KTX) is associated with a superior cardiovascular outcome, compared to other renal replacement therapies [1]. However, cardiovascular disease still represents the leading cause of death among renal transplant recipients (RTRs), and cardiovascular risk is increased by three to five times when compared with the general population [2,3]. This cannot be fully explained by the higher prevalence of conventional cardiovascular risk factors, such as diabetes, hypertension, smoking, and dyslipidemia [4]. Long-term immunosuppressive therapy and other transplantation-related risk factors, such as arterial stiffness, kidney graft function, calcium phosphate balance, and body composition may play an important role in the cardiovascular risk calculation [5,6].

### 1.2. Arterial Stiffness and Its Relevance for Cardiovascular Outcome after Kidney Transplantation

Arterial stiffness has gained in importance as a diagnostic and prognostic parameter in the management of cardiovascular disease among RTRs [5]. It is regarded as an independent predictor for cardiovascular events and mortality after KTX [7,8]. There are different methods that can be used to quantify arterial stiffness. One of them is the measurement of intra-aortic blood pressure, an invasive method that requires arterial catheterization and is, therefore, rarely used in clinical practice. Another non-invasive and more common technique is pulse wave velocity (PWV) measurement [9]. This can be measured from the arm to the ankle (brachial-ankle pulse wave velocity; baPWV), or from the carotid to the femoral artery (carotid-femoral pulse wave velocity; cfPWV) [10]. According to a consensus document of the European Society of Hypertension and the European Society of Cardiology, the measurement of the cfPWV represents the gold standard for the assessment of large-vessel arterial stiffness [11].

### 1.3. Body Composition as a Predictor for Cardiovascular Disease and Its Assessment

Moreover, the assessment of body composition has taken on a significant role in the cardiovascular evaluation of patients with chronic kidney disease (CKD). CKD can lead to extracellular volume overload and chronic uremia can cause perturbations in the electrolyte balance, as well as alterations of body composition and nutritional status [12,13,14]. Since the glomerular filtration rate (GFR) may be reduced and immunosuppressants may influence body weight, the body composition after KTX is highly variable [15,16]. Consequently, the phase angle (PhA) has gained interest for predicting cardiovascular events [17], although its biological meaning and pathogenic relevance is not yet fully understood. To date, PhA is seen as an indicator for cell membrane integrity and volume distribution between the intra- and extracellular compartments, being closely related to catabolic processes and cell death [18].

### 1.4. Immunosuppression

Although kidney function increases after transplantation, RTRs still demonstrate higher pulse wave velocities compared to healthy adults [19]. Studies show that long-term immunosuppressive therapy can have a negative impact on the cardiovascular profile. Calcineurin inhibitors (CNI) such as Cyclosporine (CsA) and tacrolimus (Tac), which have been widely used since the 1980s, represent an integral component of immunosuppression after KTX [20]. Long-term use of CNI, however, can lead to hyalinotic changes of the arteriole wall and is associated with an increased risk of hypertension, dyslipidemia, and diabetes [21,22].

Even though the calcineurin inhibitors Tac and CsA act via similar mechanisms (inhibition of the transcription of interleukin-2 and other cytokines), studies show diverging degrees of influence on arterial stiffness. Strózecki et al. investigated the differences in PWV between 76 patients undergoing therapy with Tac and 76 patients undergoing CsA therapy. They demonstrated significantly higher PWV values within the CsA group when compared with Tac (9.8 ± 2.3 versus (vs.) 8.6 ± 1.5 m/s, *p* < 0.001) [23].

Since 2011, the co-stimulation inhibitor, Belatacept (BELA), has been approved for immunosuppressive therapy after kidney transplantation in Europe. BELA is a fusion protein comprising the common Fc fragment of the human immunoglobulin G and CTLA-4 (cytotoxic T-lymphocyte-associated protein 4). CTLA-4 binds the CD (cluster of differentiation) 80 and CD 86 receptors on antigen-presenting cells and, thus, inhibits the costimulatory signals needed for T-cell activation [5].

Within the phase-III-studies, BENEFIT and BENEFIT-EXT, patients undergoing BELA therapy with more (MI) or less (LI) intensive therapy regimens were compared with patients undergoing therapy with CsA. After three years, transplant survival rates between the immunosuppressive groups were comparable, while the measured GFR was significantly higher among the BELA groups [24,25]. It could also be seen that the cardiovascular profile was superior among patients undergoing BELA therapy, compared to the CsA group, demonstrating a lower risk of hypertension, hyperlipidemia, and new-onset diabetes after transplantation (NODAT) [26]. Other studies demonstrated an improved kidney function in stable renal allograft recipients after their conversion to BELA treatment [27,28,29].

To date, only a few studies with small sample sizes have been published, investigating the differences in arterial stiffness between RTRs undergoing BELA vs. CNI therapy [30,31]. To our knowledge, there are no studies concerning the alterations of body composition under BELA therapy published. Therefore, we investigated the differences in arterial stiffness and body composition between RTRs treated with different immunosuppressants, including BELA.

## 2. Material and Methods

### 2.1. Study Population and Design

In total, 325 patients visiting the nephrological outpatient clinic at the Charité Campus Mitte between February and July 2018 for the purpose of a routine examination were included in the study. Out of 376 examined patients, 32 patients were excluded because they had not yet received a kidney transplant. In total, 19 other transplanted patients were ruled out because they were not on medication regimens with either Tac, CsA or BELA. Standard maintenance immunosuppression after renal transplantation consisted of CNI (either CsA or Tac, according to immunological risk until 2012; after 2012, tacrolimus was used as the standard), Mycophenolate and steroids, which were withdrawn in low-risk recipients. Acute infections, the inability to stand upright, arm or leg ulcers, as well as implanted defibrillators or pacemakers, represented further exclusion criteria.

**Figure 1:**
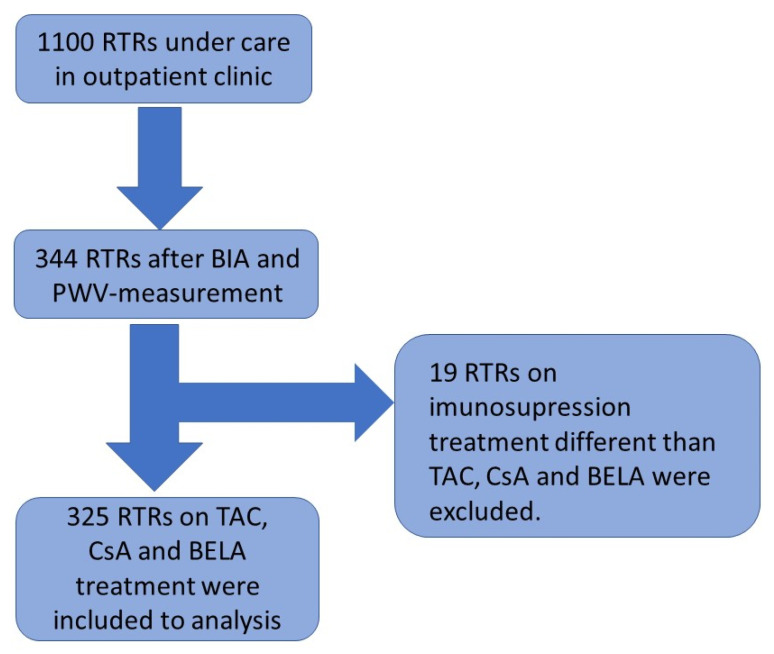
The schedule of the cross-sectional study.

The cross-sectional study of all patients included BIA and PWV measurement, as described previously (Figure 1) [32]. Demographic data, as well as information regarding the transplantation, donors, past medical history, medication, and laboratory results were extracted from the database “TBase” [33,34,35]. All investigations were conducted according to the Declaration of Helsinki. This study was approved by the local ethics committee (EA 1/252/17). Informed consent was obtained from all participants.

### 2.2. Pulse Wave Velocity Measurement

The assessment of arterial stiffness via oscillometric PWV-measurement was performed using an ankle-brachial index (ABI) device produced by boso^®^ (BOSCH + SOHN GmbH, Jungingen, Germany). The patients were requested to lie flat on their backs, with arms and legs spread to the sides, for at least five minutes before starting the measurement. After correct positioning of the cuffs, systolic and diastolic blood pressure (RR; in mmHg) was measured on both arms (2–3 cm above the antecubital fossae) and legs (1–2 cm above the ankles). In addition, ABI (systolic RR of the right or left ankle, divided by the higher systolic RR of the arms) and brachial-ankle pulse wave velocity (baPWV) (in m/s) were acquired. Carotid-femoral pulse wave velocity (cfPWV) (in m/s) and pulse pressure (PP) were calculated by the device. The following reference values were defined as physiological: RR ≤ 130/80 mmHg, PP < 50 mmHg, ABI > 0.9, baPWV < 12 m/s, cfPWV < 10 m/s [36].

### 2.3. Bioelectrical Impedance Analysis

The body composition parameters were acquired via direct segmental multi-frequency BIA, using the InBody770^®^ device (InBody Deutschland/JP Global Markets GmbH, Eschborn, Germany). With an alternating current at a power of 80 microamperes (µA) ± 10 µA, 30 impedance measurements at six different frequencies were conducted at each of the five segments (right and left arm and leg, trunk). The patients were assigned to step on the BIA scales and stand on foot electrodes for about one minute, with their arms abducted, holding two thumb electrodes. They were asked to refrain from moving or speaking. The body composition measurement comprised body water content and lean and fat mass analyses. Among other parameters, body mass index (BMI, in kilogram per square meter (kg/m^2^)), muscle (in kg) and fat mass (in kg), protein and mineral content (in grams, g), as well as skeletal body mass (in kg) and PhA (in degrees, °), were calculated. The skeletal muscle index (SMI, kg/m^2^) was calculated from body height (in m) and skeletal muscle mass. The following reference value for PhA was predefined: PhA: ≥5.5° (women), ≥6.0° (men).

### 2.4. Laboratory Analyses

All patients had fasted before giving blood samples on the examination day. Labor Berlin was responsible for the analysis of all laboratory values. Using high-performance liquid chromatography, HbA1c (in %) was determined in EDTA (ethylenediaminetetraacetic acid) blood. In addition, the C-reactive protein (CRP, in milligrams per liter (mg/L)), low-density lipoprotein (LDL) cholesterol (in milligrams per deciliter (mg/dL) and creatinine (in mg/dL) levels were measured in the serum. The estimated glomerular filtration rate (eGFR) was calculated from the CKD-EPI formula (in mL/min/1.73 m^2^). By use of an electro-chemiluminescent immunoassay (ECLIA), troponin T (in nanograms per liter (ng/L)) and NT-proBNP (N-terminal pro-B-type natriuretic peptide, in ng/L) were measured in the serum or heparin plasma. According to the official statements of Labor Berlin, the following reference values were determined: HbA1c: <6%, CRP: <5 mg/dL, LDL cholesterol: <130 mg/dL, creatinine: ≥0.51–≤0.95 mg/dL (women), ≥0.67–≤1.17 mg/dL (men), eGFR: 95–160 mL/min/1.73 m^2^ (women), 98–156 mL/min/1.73 m^2^ (men), troponin T: <50 ng/L, NT-proBNP: depending on age, between <94 ng/L (<44 years) and <526 ng/L (>74 years).

### 2.5. Statistical Analyses

Results are expressed as mean ± standard deviation for normally distributed variables or median and interquartile range (25th–75th percentile) and natural logarithm, respectively, for non-normally distributed values. Frequencies of the categorical entities were expressed as a percentage (%) and compared via a chi-square test. To analyze the differences between mean values, unpaired *t*-tests and ANOVAs (analysis of variance) were conducted. The medians were compared using a Kruskal–Wallis test. Through linear regression analysis, associations between dependent and independent continuous variables could be detected. The standardized regression coefficient was calculated, to compare the effects of independent variables on the dependent variable. By the calculation of Cohen’s d and the correlation coefficient r, the effect size of the *t*-tests could be determined. Multiple linear regression analysis allowed us to adjust for confounders and detect those variables that have an independent association with the endogenous variable.

In addition, propensity score (PS) matching was performed. The PS can be understood as the probability with which a patient receives an investigated therapy. At first, PS was calculated by multiple regression analysis, taking predefined patient characteristics into account. In a second step, a so-called 1:1-matching was conducted. Therefore, each patient from one group (i.e., BELA) was assigned to another patient from another group (i.e., Tac), with a similar PS. In this study, PS matching was performed three times (Tac-BELA, CsA-BELA, Tac-CsA). Within the matched collective, the parameters of interest were compared between the groups. All statistical analyses were performed using the program IBM SPSS Statistics 25^®^ (IBM Corp.^®^, New York, NY, USA; IBM Deutschland GmbH^®^, Ehningen, Germany). Probability values of <0.05 were considered statistically significant.

## 3. Results

### 3.1. Baseline Characteristics

Out of 325 included patients, 122 (37.5%) were female and 203 (62.5%) were male (Table 1). The patients’ mean age was 52.2 ± 13.7 years. Of these, 134 (41.9%) patients had received a living-donor transplantation, and the mean time after transplantation was 6 (2.4–11) years. In 56 (17.2%) patients, immunoglobulin (Ig)A nephropathy represented the underlying kidney disease, 52 (16%) suffered from polycystic kidney disease and 39 (12%) from glomerulonephritis (not further classified). The mean time of dialysis amounted to 4.2 (0.8–7.7) years. In total, 285 (87.7%) of the examined patients suffered from co-morbid hypertension. Arterial angiopathy (coronary artery disease (CAD) or peripheral artery disease (PAD)) was present in 68 (20.9%) of the patients. In total, 62 (19.1%) patients were diabetics and 96 (29.5%) suffered from hyperlipoproteinemia. About half of the patients (163; 50.2%) were undergoing therapy with steroids, and the mean amount of antihypertensive medication was 2 ± 1.

On the examination day, the average creatinine level was 1.7 ± 0.8 mg/dL, and the patients’ mean eGFR was 50.2 ± 20 mL/min/1.73 m^2^. Their average HbA1c was 5.5 ± 0.6% and the concentration of LDL cholesterol amounted to 123.2 ± 37.9 mg/dl. Median values for troponin T and NT-proBNP were 12 (7–22) and 288 (112–731) ng/L, respectively.

### 3.2. Comparability of the Groups

Among the study population, 211 (64.9%) patients were undergoing therapy with Tac, 73 (22.5%) patients were being treated with CsA, and 41 (12.6%) with BELA (Table 1). Prior to their conversion to BELA, the patients from the BELA group had been treated with CNI for 3.4 (1.8–6) years, on average. At the time of the examination, they had been undergoing therapy with BELA for 3.4 (2.2–4.3) years. Further information regarding the baseline and clinical characteristics of the groups is shown in Table 1. The three groups were comparable regarding most baseline characteristics, including nicotine abuse, type and time of dialysis, and most laboratory parameters, as well as their underlying kidney disease and comorbidities (Table 1). However, the groups showed some differences in age, time since transplantation, and type of kidney donation, as well as the amount of antihypertensives. Patients undergoing therapy with CsA were treated with steroids less often, while patients undergoing therapy with BELA showed significantly worse kidney function compared to patients undergoing Tac- or CsA-therapy, as many patients were converted because of poor allograft function [29,37].

### 3.3. Descriptive Statistics

#### 3.3.1. Blood Pressure and Pulse Wave Velocity Measurement

The mean systolic and diastolic blood pressure was 140.8 ± 17.6 mmHg and 85.8 ± 10.2 mmHg, respectively. The average ABI amounted to 1.08 ± 0.1 on the right and 1.12 ± 0.5 on the left side. The baPWV was 12.3 ± 2.3 m/s on the right and 12.3 ± 2.5 m/s on the left, respectively. The measurement of the cfPWV could be accomplished in 294 of the patients. On average, the patients’ cfPWV was 8.3 ± 2.3 m/s. Using ANOVA, no statistically significant differences in any of the abovementioned parameters were found between the groups (Table 2).

#### 3.3.2. Bioimpedance Analysis

The mean BMI was 25.5 ± 4.7 kg/m^2^ and the body fat percentage was 27.1 ± 9.5%, on average. The ECW/WBW ratio amounted to 0.39 ± 0.01, and the SMI was 7.67 ± 1.3 kg/m^2^, respectively. PhA could be measured in 321 of the patients and was 4.9 ± 0.9°, on average. The mean values of the mentioned parameters were comparable among the three groups. Only the ECW/WBW ratio (*p* = 0.084) showed differences, which were, however, not significant (Table 2).

#### 3.3.3. ANCOVA

After adjusting for the covariates of age, sex, BMI, logarithmical dialysis time, logarithmical time since transplantation, type of kidney donation (living-donor/deceased-donor donation), and prevalence of diabetes and creatinine levels in the serum, no significant differences in cfPWV could be found among the three therapy groups (Tac, CsA, BELA; *p* = 0.720). Age (*p* < 0.01) and the type of kidney donation (living-donor/deceased-donor donation; *p* = 0.036) showed a significant, independent association with the cfPWV using an ANCOVA. Only 28.5% of the variance could be explained by this model (r^2^ = 0.285).

In addition, no significant differences in PhA could be detected between the groups (*p* = 0.709), after adjusting for the covariates. However, a significant, independent association was found among PhA and age (*p* < 0.01), sex (*p* < 0.01), BMI (*p* < 0.01), dialysis time (*p* = 0.01) and creatinine levels (*p* < 0.01). Only, 50.1% of the variance could be explained by this model (r^2^ = 0.501).

#### 3.3.4. Propensity Score Matching

The PS model was created by a logistic regression model, adjusting for predefined patient characteristics (age, sex, BMI, dialysis time, time since transplantation, type of kidney donation, diabetes, creatinine levels) and the PS was calculated for each patient. Matching was performed three times in total (Tac-BELA, CsA-BELA, and CsA-Tac) and resulted in acceptable and comparable characteristics of predefined variables (Table 3).

At first, 30 patients undergoing Tac-therapy were matched with 30 patients undergoing BELA therapy. Mean cfPWV in the Tac- and BELA groups amounted to 9.2 ± 3.1 m/s and 8.4 ± 2.4 m/s, respectively. The difference was, however, not statistically significant (*p* = 0.287). Additionally, no difference in PhA could be found between the groups (*p* = 0.931).

In a second step, 29 patients undergoing CsA therapy were matched with 29 patients undergoing BELA therapy. Again, the mean cfPWV was 0.7 m/s higher in the CNI-based therapy group compared to the BELA group, but no statistically significant differences could be detected (*p* = 0.446). In addition, the mean PhA was similar between the groups (*p* = 0.729).

Finally, 57 patients undergoing CsA therapy were matched with 57 patients from the Tac group. The mean cfPWV and PhA were comparable and no statistically significant differences could be found between the groups (*p* = 0.697 and *p* = 0.278, respectively).

## 4. Discussion

In the present study, we could not detect any significant differences in arterial stiffness and PhA in RTRs with different immunosuppressive regimens based on CsA, Tac, or BELA. As expected, age was an essential risk factor for higher arterial stiffness. Additionally, PhA was associated with age, BMI, time of dialysis before transplantation, and kidney graft function.The reduction in the prevalence of RTRs with a cf-PWV higher than 8.1 m/s undergoing BELA treatment was reported by Melilli et al. [30] at 5 years after transplantation. Moreover, the authors revealed the correlation between arterial stiffness and age [30]. It is worth underlining that similar results were presented by Heleniak et al., where age and CVD correlated with PWV in RTRs [35]. Moreover, in the literature, an association between PWV, the recipient’s age, and dialysis vintage was shown [19,38]. All these data were similar to our results.

On the other hand, the progression of arterial stiffness in RTRs was determined by the donor’s and recipient’s ages, the blood pressure control, and the type of donor (living/cadaveric). Therefore, the older age and CV history influence the value of PWV significantly.

It was confirmed by Kolonko et al. and Kim et al. in the RTRs population and in ESRD (end-stage renal disease) patients on the waiting list for kidney transplantation [39,40]. Additionally, the authors demonstrated PWV value as a strong predictor of CVD in RTRs.

It is worth underlining that in our study, the PWV and PhA in BELA-treated patients were not better or worse in comparison to CNI. It is important to mention that prior to conversion to BELA, the patients in the BELA group had been treated with CNI for 3.4 (1.8–6) years on average. Many of those patients were converted because of CNI toxicity and had inferior renal function [29,37]. At the time of the examination, they had been undergoing therapy with BELA for 3.4 (2.2–4.3) years, and their deteriorating renal function had been stabilized. The fact that the tacrolimus group had 3–6 years’ shorter time since transplantation than the other patients would likely influence the main results in a way that might favor tacrolimus. Therefore, the propensity score analysis aimed to correct for this bias. However, data from the historic CsA cohort have to be interpreted with caution, as differences in the transplant era and other potential time-dependent variables after transplantation may have caused some undetected bias. Additionally, there were more RTRs with CVD and hyperlipoproteinemia in the BELA immunosuppressive regimen, compared to CNI. In summary, inferior renal function and comorbidities are potential factors associated with the higher cardiovascular burden in our cohort of BELA-treated patients, which might explain why a drug without a cardiovascular risk profile did not exert any beneficial effects on arterial stiffness in the study population.

Stróżecki et al. showed that CNI increased PWV significantly, compared to the non-CNI-regimen (sirolimus, mycophenolate mofetil, azathioprine) patients. Moreover, CsA influenced PWV more strongly than Tac [23]. In another study, the authors showed that CNI may contribute to vascular stiffness in RTRs, with no differences between CsA or tacrolimus [41].

There is not much data regarding the evaluation of PhA in patients after kidney transplantation. Wong et al. and Saxena et al. showed more extracellular water and lower PhA in RTRs, in comparison to healthy controls [15,42]. On the other hand, Saxena et al. indicated poorer nutritional status in participants with an eGFR of <40 mL/min, compared to eGFR ≥ 40 mL/min, based on a lower body weight, BMI, free-fat mass, and dry weight. Additionally, there were significant differences between groups in terms of PhA value, the level of extracellular water, and body cell mass.

In our study, the PhA value was slightly higher compared to that reported by Wong et al. and Saxena et al. This could be associated with the better nutritional status of RTRs. Secondly, we observed a tendency for a negative correlation between PhA and extracellular water (r = −0.17; *p* = 0.07). Finally, the mean eGFR of our patients was relatively high, at 54.1 mL/min (compatible with stage 3A chronic kidney disease), but patients on BELA have a significantly lower eGFR compared to the CNI groups. Future studies should be designed as a prospective, ideally randomized trial, with at least two measurements for each immunosuppressive regimen, to detect any differences over time in each of the groups. However, currently, no such randomized study is possible for BELA due to its limited availability.

## 5. Limitations

There are several limitations of this study that should be considered when interpreting our conclusions. First of all, this is a single-center study. The sample size was relatively small, and the study population was a heterogeneous group with different comorbidities, including cardiovascular disease, diabetes, and heart failure, different dialysis vintage, and a different time after renal transplantation. Additionally, there is obviously an important selection bias since BELA patients had received CNI for several years and had significantly lower eGFR compared to the CNI groups. We aimed to compensate for these relevant limitations by propensity score-matching, which did not reveal any statistical differences with regard to some potential confounders (Table 3). However, it has to be kept in mind that propensity score-matching may not compensate for all biases, since the matched groups are rather small, with limited statistical power. The most appropriate way to study the differential effect of Tac and CsA, or alternatively to investigate the effect of CNI withdrawal on PWV and PhA, would be a prospective randomized trial with biomarkers included before and after the change in immunosuppressive treatment.

However, despite these limitations, this study highlights some important information for RTRs in terms of arterial stiffness and PhA in this population. Hence, we cannot exclude the possibility of residual confounding. To obtain a definite answer on the effect of an immunosuppressive regimen on PWV and PhA, further studies are needed. Due to the abovementioned limitations and the limited availability of BELA, no prospective study was possible, and firm conclusions can only be drawn in a prospective randomized trial. Our data are hypothesis-generating and are important for the design of future trials in the field.

## 6. Conclusions

In our observational study, we did not observe any significant differences in arterial stiffness and PhA in RTRs under different immunosuppressive regimens. Due to the outlined limitations and a potential selection bias, further, prospective studies are necessary to definitively assess the relationship between arterial stiffness, PhA, and immunosuppressive regimen. Our data provide additional evidence to the literature on PWV and PhA in RTR and may provide the basis for future research on cardiovascular risk factors. 

## Figures and Tables

**Table 1:** Baseline characteristics.

	Entire Study Population	Tac	CsA	BELA	*p*-Value
Epidemiological and Nephrological Data					
All, *n*	325	211	73	41	
Age, years	52.2 ± 13.7	50.9 ± 13.5	54.3 ± 13	55.3 ± 15	0.056 (A)
Women, *n* (%)	122 (37.5)	79 (37.4)	27 (37)	16 (39)	0.976 (C)
Men, *n* (%)	203 (62.5)	132 (62.6)	46 (63)	25 (61)	
BMI, kg/m^2^	25.5 ± 4.7	25.4 ± 4.9	26.2 ± 4.7	25.1 ± 4	0.402 (A)
Nicotine abuse, *n* (%)	130 (40.8)	80 (38.3)	33 (46.5)	17 (43.6)	0.444 (C)
Living-donor donation, *n* (%)	134 (41.9)	97 (46.6)	21 (29.2)	16 (40)	0.034 (C)
HD, *n* (%)	200 (73.8)	126 (73.7)	49 (74.2)	25 (73.5)	0.680 (C)
PD, *n* (%)	38 (14)	24 (14)	10 (15.2)	4 (11.8)	
HD and PD, *n* (%)	32 (11.8)	21 (12.3)	1 (1.5)	5 (14.7)	
Time of dialysis, years	4.2 (0.8–7.7)	4.2 (0.8–8.2)	4.2 (1.7–7.1)	3.3 (0.8–6.1)	0.349 (B)
Time since transplantation, years	6 (2.4–11)	4.6 (1.3–7.7)	10.5 (7.8–16.2)	7.8 (5.8–11.5)	<0.01 (B)
Underlying Kidney Disease					
IgA nephropathy, *n* (%)	56 (17.2)	38 (18)	15 (20.5)	3 (7.3)	0.230 (C)
Polycystic kidney disease, *n* (%)	52 (16)	29 (13.7)	13 (17.8)	10 (24.4)	
Chronic glomerulonephritis, *n* (%)	39 (12)	25 (11.8)	11 (15.1)	3 (7.3)	
FSGS, *n* (%)	20 (6.2)	16 (7.6)	3 (4.1)	1 (2.4)	
Membranous glomerulosclerosis, *n* (%)	17 (5.2)	8 (3.8)	6 (8.2)	3 (7.3)	
Hypertensive kidney disease, *n* (%)	17 (5.2)	9 (4.3)	3 (4.1)	5 (12.2)	
Others, *n* (%)	124 (38.2)	86 (40.8)	22 (30.1)	16 (39)	
Comorbidities					
Hypertension, *n* (%)	285 (87.7)	184 (87.2)	68 (93.2)	33 (80.5)	0.133 (C)
CAD/PAD, *n* (%)	68 (20.9)	41 (19.4)	13 (17.8)	14 (34.1)	0.08 (C)
Myocardial infarction, *n* (%)	12 (3.7)	5 (2.4)	5 (6.9)	2 (4.9)	0.197 (C)
Diabetes, *n* (%)	62 (19.1)	45 (21.3)	14 (19.2)	3 (7.3)	0.113 (C)
NODAT, *n* (%)	19 (5.8)	14 (6.6)	5 (6.9)	0 (0)	0.232 (C)
Hyperlipoproteinemia, *n* (%)	96 (29.5)	60 (28.4)	22 (30.1)	14 (34.1)	0.758 (C)
Medication					
Antihypertensives, *n*	2 ± 1	2 ± 1	2 ± 2	2 ± 2	0.015 (A)
Steroids, *n* (%)	163 (50.2)	117 (55.5)	22 (30.1)	24 (58.5)	<0.01 (C)
Time on Belatacept, years				3.4 (2.2–4.3)	
Laboratory results					
eGFR, mL/min/1.73 m^2^	50.2 ± 20	54.1 ± 19	45.3 ± 18.5	38.6 ± 21.2	<0.01 (A)
Creatinine, mg/dL	1.7 ± 0.8	1.5 ± 0.6	1.8 ± 0.7	2.4 ± 1.4	<0.01 (A)
HbA1c, %	5.5 ± 0.6	5.5 ± 0.6	5.6 ± 0.5	5.4 ± 0.4	0.306 (C)
LDL cholesterol, mg/dL	123.2 ± 37.9	123 ± 38.5	123.7 ± 39.1	123.1 ± 33.4	0.991 (A)
CRP, mg/L	1.6 (0.7–5.5)	1.7 (0.7–5.2)	1.9 (0.9–6.7)	1.3 (0.5–5.6)	0.619 (B)
NT-proBNP, ng/L	288 (112–731)	286 (113–723)	315 (100–704)	269 (120–900)	1 (B)
Troponin T, ng/L	12 (7–22)	12 (7–21)	10 (6–16)	17 (7–31)	0.112 (B)

Data for continuous variables, as mean ± standard deviation or median (25th–75th percentile), for categorical variables, shown as a percentage (%). Calculation of *p*-values through ANOVA (A; normally distributed, continuous variables), the Kruskal–Wallis test (B; comparison of medians), or chi-square test (C; categorical variables). Abbreviations: Tac, tacrolimus; CsA, Cyclosporine; BELA, Belatacept; *n*, number; BMI, body mass index; HD, hemodialysis; PD, peritoneal dialysis; FSGS, focal segmental glomerulosclerosis; CAD, coronary artery disease; PAD, peripheral artery disease; NODAT, new-onset diabetes after transplantation; eGFR, estimated glomerular filtration rate; LDL, low-density lipoprotein; CRP, C-reactive protein; NT-proBNP, N-terminal pro-B-type natriuretic peptide.

**Table 2:** Results of the PWV and BIA measurements.

	Entire Study Population	Tac	CsA	BELA	*p*-Value
Pulse wave velocity Measurement					
RR rA syst., mmHg	140.8 ± 17.6	139.4 ± 17.3	144.5 ± 17.1	141.5 ± 19.7	0.140
RR rA diast., mmHg	85.8 ± 10.2	85.3 ± 9.4	88.1 ± 11.3	84.3 ± 11.7	0.120
ABI right	1.08 ± 0.1	1.09 ± 0.1	1.07 ± 0.1	1.06 ± 0.1	0.168
ABI left	1.12 ± 0.5	1.15 ± 0.7	1.06 ± 0.1	1.1 ± 0.1	0.516
baPWV right, m/s	12.3 ± 2.3	12.1 ± 2.2	12.8 ± 2.8	12.2 ± 2	0.170
baPWV left, m/s	12.3 ± 2.5	12.2 ± 2.3	12.6 ± 2.8	12.4 ± 3	0.633
cfPWV, m/s	8.3 ± 2.3	8.2 ± 2.1	8.6 ± 2.6	8.4 ± 2.4	0.410
Bioelectrical Impedance Analysis					
BMI, kg/m^2^	25.5 ± 4.7	25.4 ± 4.9	26.2 ± 4.7	25.1 ± 4	0.402
BF, %	27.1 ± 9.5	26.8 ± 9.7	28.7 ± 8.9	25.4 ± 9	0.160
EZW/GKW	0.39 ± 0.01	0.389 ± 0.01	0.39 ± 0.01	0.393 ± 0.01	0.084
SMI, kg/m^2^	7.67 ± 1.3	7.62 ± 1.3	7.67 ± 1.3	7.91 ± 1.2	0.407
PhA, °	4.9 ± 0.9	4.9 ± 0.8	4.8 ± 0.9	4.7 ± 0.9	0.268

Data for continuous variables as mean ± standard deviation. Calculation of *p*-values through ANOVA (normally distributed, continuous variables). Abbreviations: Tac, tacrolimus; CsA, Cyclosporine; BELA, Belatacept; RR rA syst., systolic blood pressure measured on the right arm; RR rA diast., diastolic blood pressure measured on the right arm; ABI, ankle-brachial index; baPWV, brachial-ankle pulse wave velocity; cfPWV, carotid-femoral pulse wave velocity; BMI, body mass index; BF, body fat; ECW/WBW, extracellular water and whole-body water ratio; SMI, skeletal muscle index; PhA, phase angle.

**Table 3:** Propensity score matching.

	Tac	BELA	*p*-Value	CsA	BELA	*p*-Value	CsA	Tac	*p*-Value
Matched patients, *n*	30	30		29	29		57	57	
Age, years	53.4 ± 11.4	55.8 ± 14.4	0.472 (A)	56.2 ± 12.8	54.6 ± 14.3	0.651 (A)	54.5 ± 13.7	52 ± 12.7	0.302 (A)
Women, *n* (%)	10 (33.3)	9 (30)	0.781 (C)	10 (34.5)	9 (31)	0.780 (C)	21 (36.8)	13 (22.8)	0.101 (C)
Men, *n* (%)	20 (66.6)	21 (70)		19 (65.5)	20 (69)		36 (62.2)	44 (77.2)	
BMI, kg/m^2^	25.6 ± 4.3	25.1 ± 3.7	0.629 (A)	25.7 ± 4.1	25 ± 3.8	0.446 (A)	25.7 ± 4	25.9 ± 4.7	0.772 (A)
Time of dialysis, years	3.8 (0.2–7.3)	2.8 (0.8–6.1)	0.824 (B)	3.9 (1.2–5.8)	3 (0.7–6.3)	0.994 (B)	4.8 (1.7–6.8)	5.3 (1.5–7.8)	0.713 (B)
Time since transplantation, years	8.7 (4.3–16)	6.2 (5.8–10.4)	0.947 (B)	9 (7.6–12.3)	8.3 (5.9–12.3)	0.969 (B)	9.1 (7.3–19.7)	10.4 (5.8–18.6)	0.986 (B)
Living-donor donation, *n* (%)	10 (33.3)	12 (40)	0.592 (C)	8 (27.6)	10 (34.5)	0.57 (C)	17 (29.8)	21 (36.8)	0.427 (C)
Diabetes, *n* (%)	1 (3.3)	2 (6.7)	0.554 (C)	4 (13.8)	2 (6.9)	0.389 (C)	12 (21.1)	12 (21.1)	1 (C)
Creatinine, mg/dL	1.91 ± 0.91	1.95 ± 0.86	0.848 (A)	2.03 ± 0.8	2.06 ± 1.04	0.722 (A)	1.72 ± 0.6	1.76 ± 0.8	0.783 (A)
PhA, °	4.8 ± 0.9	4.8 ± 0.9	0.931 (A)	4.8 ± 0.8	4.8 ± 0.9	0.729 (A)	4.8 ± 0.9	4.9 ± 0.9	0.278 (A)
cfPWV, m/s	9.2 ± 3.1	8.4 ± 2.4	0.287 (A)	9.1 ± 3.2	8.4 ± 2.6	0.446 (A)	8.6 ± 2.6	8.4 ± 2.5	0.697 (A)

Data for continuous variables as mean ± standard deviation or median (25th–75th percentile), for categorical variables as a percentage (%). Calculation of *p*-values through ANOVA (A; normally distributed, continuous variables), Kruskal–Wallis test (B; comparison of medians), or chi-square test (C; categorical variables). Abbreviations: Tac, tacrolimus; BELA, Belatacept; CsA, Cyclosporine; *n*, number; BMI, body mass index; PhA, phase angle; cfPWV, carotid-femoral pulse wave velocity.
